# Thyroid Function Affects the Risk of Post-stroke Depression in Patients With Acute Lacunar Stroke

**DOI:** 10.3389/fneur.2022.792843

**Published:** 2022-03-03

**Authors:** Jianglong Guo, Jinjing Wang, Yue Xia, Shiyi Jiang, Pengfei Xu, Chunrong Tao, Wen Sun, Xinfeng Liu

**Affiliations:** ^1^Stroke Center and Department of Neurology, The First Affiliated Hospital of USTC, Division of Life Sciences and Medicine, University of Science and Technology of China, Hefei, China; ^2^Department of Neurology, Affiliated Jinling Hospital, Medical School of Nanjing University, Nanjing, China

**Keywords:** thyroid dysfunction, TSH, post-stroke depression, cerebral small vessel disease, stroke

## Abstract

**Objective:**

This study aimed to investigate whether thyroid function profiles are associated with post-stroke depression (PSD) and evaluate the mediation effect of cerebral small vessel disease (cSVD) on the association of thyroid function profiles and PSD in patients with acute ischemic lacunar stroke.

**Methods:**

In this study, 372 patients with confirmed acute ischemic lacunar stroke within 3 days of onset were consecutively recruited. Serum levels of thyroid hormones and thyroid antibodies were detected on admission. Lacunar infarcts, white matter lesions, cerebral microbleeds, and enlarged perivascular spaces were rated using validated scales. The severity of depression was scored with the 24-item Hamilton Depression Scale in the hospital after a week of stroke onset. Multivariate regression was utilized to analyze the association of thyroid function profiles and PSD. Mediation analysis was employed to evaluate the effect of cSVD on the association of thyroid function profiles and PSD.

**Results:**

A total of 87 (23.4%) participants were diagnosed with depression after stroke. Serum thyroid-stimulating hormone (TSH) levels were significantly higher in patients with PSD than in those without PSD, while free triiodothyronine (FT3) and free thyroxine (FT4) were not significantly different between the two groups. After adjusting for potential confounders, serum TSH levels were positively associated with the risk of PSD (OR = 1.228; 95% CI: 1.053–1.431, *p* = 0.009). A similar association was also found between the total cSVD burden score and PSD (OR = 2.137; 95% CI: 1.634–2.793, *p* < 0.001). Further mediation analysis indicated that 26.37% of the association between TSH and PSD was mediated by cSVD.

**Conclusions:**

Serum TSH levels on admission can probably predict depression after acute ischemic lacunar stroke.

## Introduction

Stroke is the second leading cause of death and the third leading cause of disability worldwide (1–3). Most survivors are vulnerable to neuropsychological complications, including depression (4), anxiety (5), fatigue (6, 7), dementia (8), apathy (9), and insomnia (10). Post-stroke depression (PSD) is the most common subtype and commonly refers to a depressive state after stroke onset (11). The frequency of PSD has been investigated in many studies worldwide. Different studies always concluded different results. Approximately one-third of patients who had stroke are affected by PSD (12). The existence of PSD not only brings a heavy financial burden to individuals but also hinders the process of stroke rehabilitation.

Thyroid hormones are essential for brain development and are also important factors to warrant brain functions throughout life (13). It has been shown that during the reorganization of the brain after a stroke, brain development mechanisms may be reactivated and involve cascades regulated by thyroid hormones (14). Moreover, a considerable number of studies have indicated that thyroid dysfunction is associated with the risk of stroke (15) as well as adverse stroke outcomes (13, 16). Additionally, the relationship between thyroid function and depression has long been recognized (17). Patients with thyroid disease are more likely to have depression symptoms. In contrast, depression may be accompanied by various subtle thyroid abnormalities. Thus, we speculate that thyroid dysfunction is also associated with PSD.

With the development of medical imaging technology, cerebral small vessel disease (cSVD) has been widely studied. cSVD is used to describe a group of pathological processes that affect small vessels of the brain (18). On the one hand, an increasing number of evidence links cSVD to PSD (19, 20). Zhang et al. conducted a magnetic resonance imaging-based cohort study and demonstrated that the total magnetic resonance imaging burden of cSVD is associated with PSD in patients who had stroke (19). Another study including 294 patients revealed that cSVD is associated with late-onset depression and increases the risk for PSD (20). On the other hand, investigators demonstrated that cSVD was also associated with thyroid dysfunction (21). All these results raise the question of whether cSVD can be regarded as a mediating factor in the relationship between thyroid dysfunction and PSD.

Therefore, our purpose was to investigate the association between thyroid dysfunction and PSD and to evaluate the effect of cSVD on the association in patients with acute ischemic lacunar stroke.

## Methods

### Participants

Patients with first-ever acute ischemic lacunar stroke hospitalized at the First Affiliated Hospital of USTC were included from September 2019 to July 2021. Lacunar stroke was defined as an acute stroke syndrome with a round or ovoid, subcortical, small deep infarct (diameter < 15 mm) based on diffusion-weighted imaging (DWI). If there were no visible lesions on baseline MRI, we employed pre-defined clinical criteria for specific clinical lacunar syndromes (22). Each patient was subjected to a series of examinations, including neurological investigations, blood tests, and MRI scans. We used the National Institutes of Health Stroke Scale (NIHSS) to assess the stroke severity of patients on admission. The inclusion criteria were as follows: (1) patients older than 18 years; (2) first-ever acute lacunar stroke; (3) within 3 days of stroke onset. Patients with (1) pre-existing thyroid diseases (such as thyroid inflammation, Goiter, Thyroid tumors, etc.); (2) communication barriers; (3) presence of cancer or other chronic diseases that easily cause mood disorders; (4) a cardioembolic source of stroke; (5) over 50% stenosis of the affected large artery; (6) other stroke types and unexplained strokes type were excluded. We also excluded those with a history of depression or on any medication that could affect emotional status or thyroid functions (such as antidepressants, estrogen, androgen, and glucocorticoids) (23). Written informed consent was obtained from all participants. The study protocol was approved by the local Ethical Committee on Human Experimentation, and our clinical registration number was ChiCTR2100043886.

### Measurements of Thyroid Hormones

Blood samples for thyroid function tests were collected within 24 h after admission. The serum was separated from blood and then stored at −80°C. Standardized radioimmunoassay kits (Beckman Coulter, USA) were employed to measure serum levels of thyroid-stimulating hormone (TSH), free triiodothyronine (FT3), and free thyroxine (FT4). Additionally, anti-thyroglobulin antibodies (TGAb) and thyroid microsomal antibody (TMAb) were also detected.

### Measurements of Depression

Diagnostic and Statistical Manual of Mental Disorders (DSM-V) criteria were used for the diagnosis of depression for all patients, and the severity of depression was scored by the 24-item Hamilton Depression Scale (HAMD-24) (24) in the hospital after a week of stroke onset. Two clinicians made the diagnosis of depression according to DSM-V, and they did not know the thyroid hormone information of all patients who had stroke. The HAMD-24 has been widely used by investigators due to its proven reliability and validity among patients who had stroke (25, 26). It includes 24 items, and each item is scored from 0 to 2 or 0 to 4. Finally, the scores for each item were summed to obtain the total score. The highest total score indicates the most severe depression. In addition, the Chinese version of the Lubben Social Network Scale (27) was also employed in evaluating interpersonal communication.

### Neuroimaging Assessment

All patients underwent a 3-T brain MRI scan (Signa HDe, General Electric Company, Fairfield, CT, USA) within 7 days after admission. Sequences included T1-weighted, T2-weighted, DWI, fluid-attenuated inversion recovery (FLAIR), and axial susceptibility-weighted imaging (SWI).

There are four main kinds of imaging markers of cSVD: white matter hyperintensities (WMH), lacunar infarcts (Lis), cerebral microbleeds (CMBs), and enlarged perivascular spaces (EPVSs) (28–31). In brief, CMBs were defined as small (diameter < 10 mm) homogenous rounded lesions of signal loss on SWI (32). Both periventricular WMH and deep WMH were graded using the Fazekas scale from 0 to 3 on FLAIR images (33). Extensive WMHs referred to deep white matter ≥ 2 score or periventricular white matter with 3 score. Enlarged perivascular space (EPVS) was identified as round, oval, or linear-shaped lesions with signal intensity equal to the cerebrospinal fluid and without a T2-hyperintense rim on FLAIR imaging (34). They were hyperintensities on T2-weighted images and were rated in the basal ganglia or centrum semiovale. We used a three-category ordinal scale to assess the severity of EPVS in the basal ganglia or centrum semiovale (mild, 0–10; moderate, 11–25; extensive, >25).

According to neuroimaging standards for cSVD (34), the total burden of cSVD ranging from 0 to 4 was calculated by accumulating four markers of cSVD. Each marker of cSVD was awarded 1 point based on the following principle: (1) presence of Lis, (2) presence of CMBs, (3) extensive WMLs, and (4) moderate to extensive EPVS (30).

### Statistical Analyses

Normal distribution of the study data was detected by the Kolmogorov–Smirnov test. Continuous variables are expressed as the means (*SD*) or median (IQR) as appropriate. Categorical variables are expressed as numbers (percentages). The differences between the PSD group and the non-PSD group were compared using Student's *t*-test or Mann–Whitney's *U*-test for continuous variables and χ^2^-test or Fisher exact test for categorical variables. Logistic regression was employed to assess the association between thyroid hormone levels and PSD. In addition, we performed mediation analysis to examine the mediating effect of cSVD on the association between serum TSH level and the risk of PSD. Serum TSH level was a predictor variable (X); total burden of cSVD was a mediator (M); PSD was an outcome variable (Y). The bootstrap method was used to test the mediation effect. A *p*-value of 0.05 was considered statistically significant. All data were analyzed with SPSS software (SPSS for Windows, version 23).

## Results

### Baseline Characteristics of Study Participants

**Figure 1:**
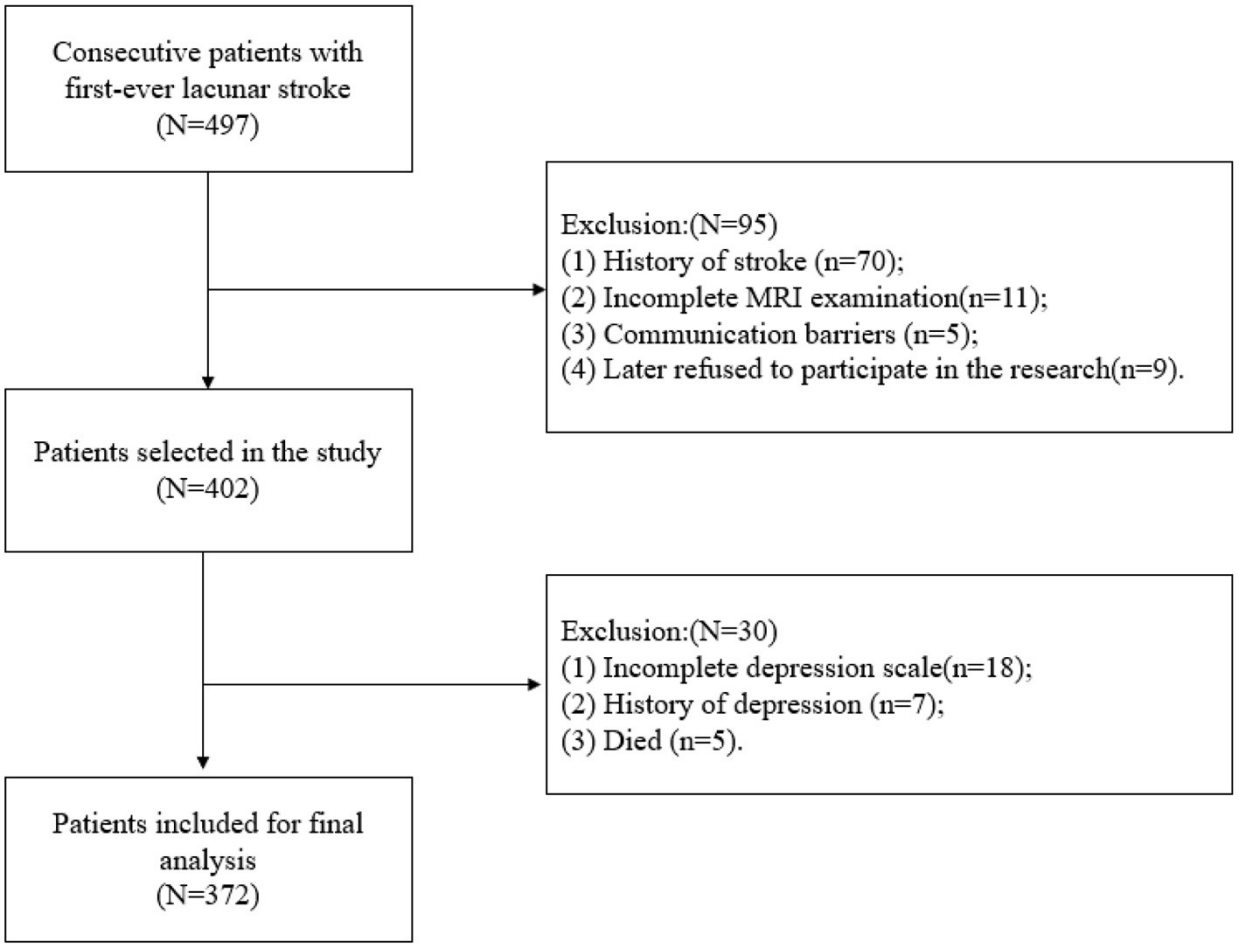
Flow chart of patient inclusion.

As presented in Figure 1, we finally enrolled a total of 372 participants in the present study, 87 (23.4%) of whom were diagnosed with PSD. The mean age of the participants was 58.6 ± 12 years. The demographic data and thyroid function profiles are shown in Table 1. No significant difference was found in sex, hypertension, diabetes mellitus (DM), smoking, body mass index (BMI), serum glucose (GLU), hemoglobin (HB), or total cholesterol (TC) between the PSD group and the non-PSD group. However, the two groups showed significant differences in terms of age (*p* = 0.043), NIHSS score (*p* = 0.002), C-reactive protein (CRP) (*p* = 0.002), uric acid (UA) (*p* = 0.006), T3 (*p* = 0.043), TSH (*p* = 0.027), and Lubben score (*p* = 0.002).

**Table 1:** Baseline characteristics of study participants without and with PSD.

**Characteristics**	**All study patients (*n* = 372)**	**Non-PSD (*n* = 285)**	**PSD (*n* = 87)**	***p-*** **value**
Age, mean (SD), y	58.6 (12.0)	57.9 (12.7)	60.9 (9.2)	0.043^*^
Female *n* (%)	117 (31.5)	84 (29.5)	33 (37.9)	0.137
Hypertension, *n* (%)	246 (66.1)	177 (62.1)	69 (79.3)	0.245
DM *n* (%)	114 (30.6)	81 (28.4)	33 (37.9)	0.092
Smoking, *n* (%)	135 (36.3)	105 (36.8)	30 (34.5)	0.689
NIHSS score, median (IQR)	2.0 (1.0–4.0)	2.0 (0–4.0)	2.0 (2.0–4.0)	0.002^*^
BMI, mean (SD), kg/m^2^	25.0 (3.3)	24.8 (3.2)	25.6 (3.4)	0.058
GLU, median (IQR), mmol/L	5.1 (4.7–6.4)	5.1 (4.7–6.8)	5.5 (4.9–6.0)	0.984
HB median (IQR), g/L	137.0 (128.0–149.8)	138.0 (128.0–150.0)	137.0 (131.0–146.0)	0.503
CRP, median (IQR), mg/L	1.4 (0.5–2.8)	1.3 (0.5–2.6)	1.7 (1.0–3.5)	0.002^*^
UA mean (SD), μmol/L	307.1 (104.8)	313.3 (100.3)	286.9 (116.7)	0.006^*^
TC, mean (SD), μmol/L	4.2 (1.2)	4.2 (1.1)	4.3 (1.3)	0.469
T3, median (IQR), pmol/L	1.2 (1.0–1.4)	1.2 (1.0–1.4)	1.2 (1.0–1.3)	0.043^*^
T4, median (IQR), pmol/L	110.4 (94.4–127.4)	110.8 (94.7–126.6)	103.8 (94.0–130.5)	0.855
FT3, median (IQR), pmol/L	4.5 (4.2–4.7)	4.5 (4.2–4.8)	4.3 (4.1–4.6)	0.055
FT4, median (IQR), pmol/L	11.2 (9.9–12.6)	11.3 (10.0–12.5)	10.3 (9.6–13.3)	0.655
TSH, median (IQR), mIU/L	2.0 (1.2–3.1)	2.0 (1.2–3.1)	2.3 (1.4–3.2)	0.027^*^
TMAb, median (IQR), IU/mL	5.0 (2.4–9.8)	4.5 (2.4–10.6)	6.9 (1.8–9.6)	0.195
TGAb, median (IQR), IU/mL	8.7 (5.2–15.3)	8.6 (5.2–11.8)	9.1 (3.9–15.7)	0.173
HAMD score, median (IQR)	4.0 (2.0–6.0)	3.0 (1.0–5.0)	14.0 (10.0–20.0)	0.000^*^
Lubben score, median (IQR)	29.0 (18.0–37.0)	28.0 (17.0–37.0)	32.0 (26.0–40.0)	0.002^*^
LIs, *n* (%)	236 (63.4)	171 (60.0)	64 (73.6)	0.025^*^
CMBs, *n* (%)	143 (38.4)	113 (39.6)	30 (34.5)	0.386
Periventricular WMH, median (IQR), score	1.0 (1.0–2.0)	1.0 (1.0–2.0)	1.0 (1.0–2.0)	0.134
Deep WMH, median (IQR), score	1.0 (0–2.0)	1.0 (0–2.0)	1.0 (0–2.0)	0.109
Centrum semiovale EPVS, median (IQR), score	3.0 (2.0–4.0)	3.0 (2.0–4.0)	4.0 (2.0–4.0)	0.870
Basal ganglia EPVS, median (IQR), score	3.0 (2.0–4.0)	3.0 (1.0–4.0)	3.0 (2.0–4.0)	0.017^*^
Total burden of cSVD, median (IQR), score	2.0 (1.0–3.0)	2.0 (1.0–3.0)	2.0 (2.0–3.0)	0.031^*^

*PSD, post-stroke depression; SD, standard deviation; IQR, interquartile range; DM, diabetes mellitus; NIHSS, national institutes of health stroke scale; BMI, body mass index; GLU, serum glucose; HB, hemoglobin; CRP, C-reactive protein; UA, uric acid; TC, total cholesterol; T3, triiodothyronine; T4, thyroxine; FT3, free triiodothyronine; FT4, free thyroxine; TSH, thyroid-stimulating hormone; TMAb, thyroid microsomal antibody; TGAb, thyroid globulin antibody; HAMD, hamilton depression scale; Lis, lacunar infarcts; CMBs, cerebral microbleeds; WMH, white matter hyperintensity; EPVS, enlarged perivascular spaces; cSVD, cerebral small vessel disease*.

*The p-value for the differences between patients without PSD and PSD*.

* *Means p < 0.05*.

### Associations Between Thyroid Hormones and PSD

Table 2 reveals the association between each thyroid hormone and PSD. Univariate analysis suggested that serum T3 and TSH levels were associated with an increased risk of PSD. However, multivariate regression results after adjusting for age and sex indicated that only serum TSH level was positively related to PSD, with an adjusted OR of 1.168 (95% CI, 1.016–1.341; *P* = 0.029). After adjusting for all the potential confounders in model 2, TSH remained an independent predictor of PSD with an adjusted OR of 1.228 (95% CI, 1.053–1.431; *P* = 0.009).

**Table 2:** Multivariate logistic regression analysis between each thyroid hormone and PSD.

**Parameter**	**Multivariate**							
	**Model 1**				**Model 2**			
	**OR**	**95% CI**		***p***	**OR**	**95% CI**		***p***
TSH	1.168	1.016	1.341	0.029^*^	1.228	1.053	1.431	0.009^*^
T3	0.422	0.157	1.136	0.088	0.708	0.245	2.043	0.523
T4	0.998	0.987	1.010	0.739	0.998	0.986	1.010	0.733
TGAb	0.996	0.976	1.016	0.693	0.995	0.973	1.017	0.652
TMAb	1.006	0.995	1.017	0.308	1.006	0.995	1.018	0.275

*Model 1: adjusted for Age and Sex*.

*Model 2: model 1 + diabetes mellitus, NIHSS, BMI, CRP, UA, LUBBEN*.

*T3, triiodothyronine; T4, thyroxine; TSH, thyroid-stimulating hormone; TMAb, thyroid microsomal antibody; TGAb, thyroid globulin antibody; CI, confidence interval; OR, odds ratio*.

* *Means p < 0.05*.

### Associations Between cSVD and PSD

We also conducted a multivariate logistic regression analysis to assess the association between cSVD and PSD. Among these imaging markers of cSVD, Lis, CMB, and basal ganglia EPVS were significantly associated with PSD (Table 3). After adjusting for age and sex (Model 1), the total burden of cSVD was a risk factor for PSD (OR = 2.079, 95% CI, 1.619–2.671; *P* < 0.001). Furthermore, after adjusting for more confounders, the association between the total burden of cSVD and PSD remained statistically significant (OR = 2.137, 95% CI, 1.634–2.793; *P* < 0.001).

**Table 3:** Multivariate logistic regression analysis between the cSVD and PSD.

**Parameter**	**Multivariate**							
	**Model 1**				**Model 2**			
	**OR**	**95% CI**		***p***	**OR**	**95% CI**		***p***
LIs	2.664	1.466	4.841	0.001^*^	2.254	1.200	4.235	0.012^*^
CMBs	1.815	1.108	2.975	0.018^*^	1.981	1.169	3.357	0.011^*^
Periventricular WMH	1.149	0.882	1.498	0.304	1.139	0.856	1.515	0.373
Deep WMH	1.116	0.862	1.444	0.404	1.043	0.794	1.370	0.765
Centrum semiovale EPVS	1.001	0.808	1.240	0.994	1.043	0.831	1.310	0.714
Basal ganglia EPVS	1.311	1.038	1.655	0.023^*^	1.392	1.088	1.781	0.008^*^
Total burden of cSVD	2.079	1.619	2.671	0.000^*^	2.137	1.634	2.793	0.000^*^

*Model 1: adjusted for Age and Sex*.

*Model 2: model 1 + diabetes mellitus, NIHSS, BMI, CRP, UA, LUBBEN*.

*Lis, lacunar infarcts; CMBs, cerebral microbleeds; WMH, white matter hyperintensity; EPVS, enlarged perivascular spaces; cSVD, cerebral small vessel disease; CI, confidence interval; OR, odds ratio*.

* *Means p < 0.05*.

### Mediation Analysis of Total cSVD Burden on the Association Between TSH and PSD

**Figure 2:**
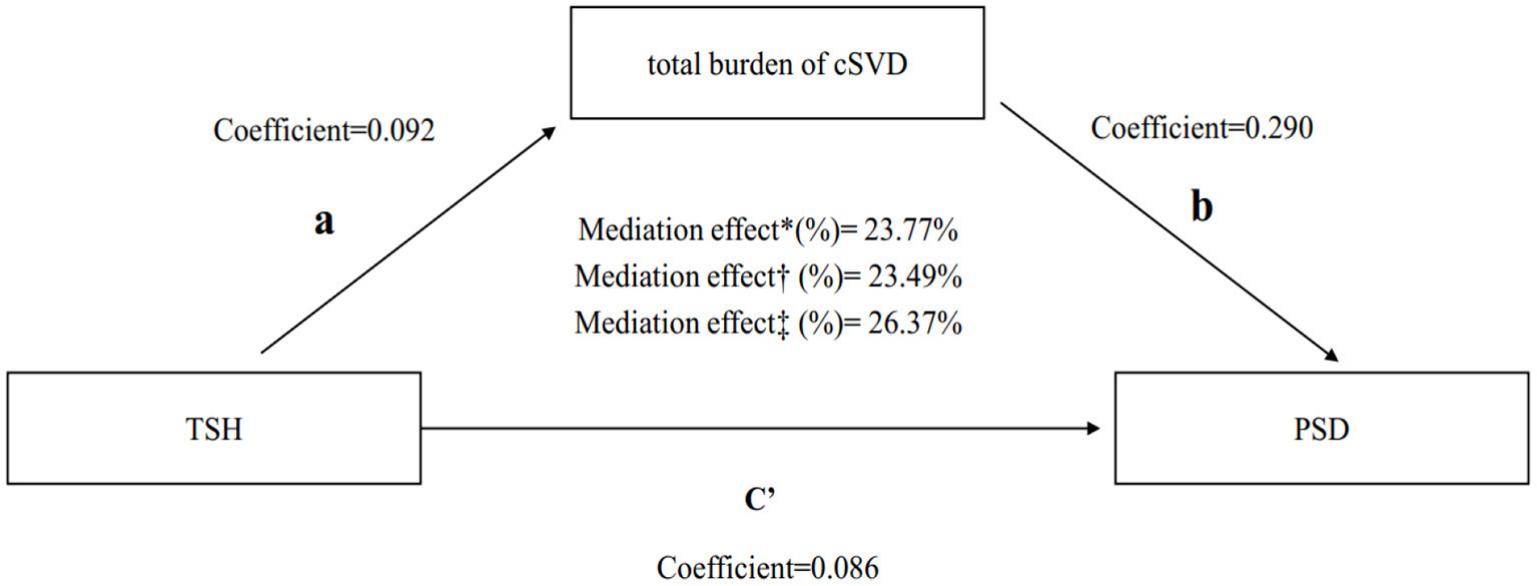
Mediation analysis of total cSVD burden on the association between TSH and PSD. ^*^Unadjusted.^†^Adjusted by module 1.^‡^Adjusted by module 2. TSH, thyroid stimulating hormone; PSD, post-stroke depression; cSVD, cerebral small vessel disease.

A significant partial mediation effect for total cSVD burden on PSD was observed. The indirect effect was 0.125 (0.011–0.275), direct effect was 0.35 (0.048–0.629), and total effect was 0.475 (0.095–0.856) (Table 4). Thus, the percentage of the total effect on the TSH-PSD association mediated by the total cSVD burden was estimated at 26.37% (Figure 2).

**Table 4:** Mediation analysis of the effect of the total cSVD burden on the PSD.

	**Indirect effect (BootLLCI-BootULCI)**	**Direct effect (BootLLCI-BootULCI)**	**Total effect (BootLLCI-BootULCI)**	**Mediation effect (%)**
Unadjusted	0.099 (0.004–0.212)	0.318 (−0.007–0.651)	0.417 (0.04–0.792)	23.77
Model 1	0.094 (−0.013–0.235)	0.307 (−0.012–0.625)	0.402 (0.023–0.781)	23.49
Model 2	0.125 (0.011–0.275)	0.350 (0.048–0.629)	0.475 (0.095–0.856)	26.37

*Model 1: adjusted for Age and Sex*.

*Model 2: model 1 + diabetes mellitus, NIHSS, BMI, CRP, UA, LUBBEN*.

## Discussion

In the present study, we aimed to investigate the association between thyroid hormone levels and PSD at the acute phase of acute lacunar stroke. Our findings suggested that only serum TSH was an independent predictor of PSD. Additionally, mediation analyses indicated that cSVD was a mediating factor in the association between TSH levels and PSD.

Although the association between TSH and depression has been extensively investigated, studies have been conducted in population groups with different diseases. A Danish population-based study indicated that suppressed TSH seems to be associated with the risk of subclinical depression (35). However, a study in patients with autoimmune disease showed that TSH levels were not different in patients with or without depression (36). In contrast, a considerable amount of available evidence elucidated that the prevalence of depressive symptoms in patients with hypothyroidism or subclinical hypothyroidism (SCH) is higher (37). A study including 216 patients with Graves' disease revealed that patients with SCH might have an increased risk of depression (38). Consistent with previous research, our results showed the same trend in patients who had acute ischemic lacunar stroke.

Research into PSD has blossomed over the past 2 decades. Previous studies (19, 21) established that cSVD was associated with both thyroid function and PSD, which was similar to the results of our research. However, we further analyzed the relationship among cSVD, thyroid function, and PSD and believed that there was a mediating effect among them. In addition, the extent to which it is mediated by cSVD has been poorly investigated. Our results showed that 26.37% of the association between TSH and PSD was mediated by cSVD. Our study might have two contributions to the field. First, our findings suggested that serum TSH levels on admission might predict depression after acute ischemic lacunar stroke. The risk factors associated with PSD would help to identify the disease and intervene in advance. On the other hand, it is an attempt to further explore the underlying mechanism of PSD. To the best of our knowledge, this is the first study to explore the role of cSVD in the association between TSH levels and PSD.

Previously, investigators proposed the “brain hypothyroidism” hypothesis to explain the association between TSH and PSD (39). The theory believes that depression is a state of local hypothyroidism in the brain that can be attributed to brain type II deiodinase inhibition as well as impaired transport of T4 across the blood-brain barrier (BBB). However, the theory cannot explain the role of cSVD in the association between TSH and PSD. Researchers later found that deficiency of thyroid hormone can also impair blood vessel function (40). Subclinical hypothyroidism can impair the relaxation of vascular smooth muscle cells, leading to increased systemic vascular resistance and arterial stiffness. This explained the role of cSVD in the association between TSH and PSD. It also provides evidence to support the use of cSVD as a mediating factor to study the relationship between thyroid function and PSD. This is another step forward for understanding the potential mechanism of PSD.

Previous studies (16, 41) usually divide patients into different subgroups, such as the subclinical hypothyroidism group, subclinical hyperthyroidism group, and euthyroidism group, according to reference ranges of serum TSH concentrations and other thyroid hormone concentrations. However, the reference limits of serum TSH concentrations remain controversial (42). For instance, an American study reported the normal reference ranges of serum TSH levels as 0.45–4.5 mIU/L based on the statistically defined reference range (43), whereas another German study based on ultrasound examination defined a normal TSH range of 0.3–0.7 mIU/L (44). Obviously, the range of normal TSH varies from laboratory to laboratory. This in part can be attributed to age, sex, acute illnesses, diurnal variations, and ethnicity (42). In this work, we did not use these reference limits of thyroid hormones but directly used quantitative data for analysis to avoid the abovementioned problems.

In our research, confounding factors were controlled as much as possible. The well-known confounders include age, sex, NIHSS, LUBBEN, diabetes mellitus, CRP, and UA (45, 46). In addition, we also included BMI as a potential confounder because serum TSH levels are elevated in overweight and obese individuals, which may erroneously indicate subclinical hypothyroidism (47). However, we did not take all the possible confounders into consideration, especially iodine intake. Research has found that high iodine intake is a risk factor for overt hypothyroidism. Subclinical hypothyroidism is more common in areas with relatively iodine-rich: 6.1–18.0%, while iodine-deficient areas are 0.9–3.8% (48). Differences in iodine intake will affect research results. Consequently, iodine intake should be considered a potential confounder in further studies.

### Limitations and Recommendations

There are certain limitations that should be discussed. First, only the acute phase study was conducted in our study, and follow-up data for the subacute and chronic phases were lacking. Hence, future research should investigate the association of TSH and PSD during subacute and chronic phases. Second, the sample size in our study was relatively small. Only 372 patients who had lacunar stroke were recruited for analysis. Thus, large cohort studies should be performed in the future. Third, patients presenting serious cognitive and communication barriers in the study were excluded, so the sample cannot fully represent all patients who had lacunar stroke. It is a fact that we cannot exclude that lower TSH levels were already present before stroke onset. According to previously published data, the incidence of subclinical hyperthyroidism is no more than 0.5% (49). Despite that, we could still not preclude potential selection bias. This is also a limitation of the article.

In brief, this was an attempt to evaluate the association of thyroid hormones and PSD among patients with lacunar stroke. Future research should consider the following aspects: (1) to confirm the exact association between thyroid hormone levels and PSD in all patients who had stroke; (2) PSD also needs further investigation to determine its association with thyroid axis hormones and other components of the limbic system; and (3) in addition to cSVD, whether there are other factors that also play a mediating role.

## Conclusion

In conclusion, the investigation demonstrated that serum TSH levels on admission can probably predict depression after acute ischemic lacunar stroke. In addition, our findings highlighted that cSVD played a mediating role in the association of TSH and PSD. Thus, combined TSH and cSVD may be a reasonable and useful approach for the assessment and prevention of PSD in the future.

## Data Availability Statement

The raw data supporting the conclusions of this article will be made available by the authors, without undue reservation.

## Ethics Statement

The studies involving human participants were reviewed and approved by the Ethics Committee of the First Affiliated Hospital of USTC. The patients/participants provided their written informed consent to participate in this study.

## Author Contributions

JG was responsible for execution of the research project, data analysis, and writing of the manuscript. JW was responsible for execution of the research project and review and critique of the manuscript. YX and SJ were responsible for execution of the research project. PX was responsible for review and critique of the manuscript. CT was responsible for data analysis of the manuscript. WS was responsible for conception, organization, design of the statistical analysis, and review and critique of the manuscript. XL was responsible for conception, organization, review and critique of the manuscript, and securing funding. All authors contributed to the article and approved the submitted version.

## Funding

This work was supported by National Natural Science Foundation of China (U20A20357), Natural Science Foundation of Anhui Province (2108085MH271), and the Fundamental Research Funds for the Central Universities (WK9110000056).

## Conflict of Interest

The authors declare that the research was conducted in the absence of any commercial or financial relationships that could be construed as a potential conflict of interest.

## Publisher's Note

All claims expressed in this article are solely those of the authors and do not necessarily represent those of their affiliated organizations, or those of the publisher, the editors and the reviewers. Any product that may be evaluated in this article, or claim that may be made by its manufacturer, is not guaranteed or endorsed by the publisher.
